# Notices to the Clergy and Ministers of Religion

**Published:** 1887-06-04

**Authors:** 


### NOTICES TO THE CLERGY AND MINISTERS OF RELIGION.

The Proprietors of The Hospital will send copies of this Special Supplement, on application, to any clergyman or minister of religion, who may require them for distribution amongst the more wealthy members of each congregation. They will be supplied, post free, at three-halfpence per copy, or, if sent for to 30, Sardinia Street, W.C., in bundles of not less than twenty-six copies, at the usual trade price, viz., one shilling and fivepence per twenty-six copies, or five shillings and eightpence per four quires (one hundred and four copies).

It is requested that an early notification may be sent to the Editor of The Hospital of all special sermons to be preached in aid of the hospitals at any of the services on Hospital Sunday, June 19th. All such notices should be addressed to the Editor of The Hospital, Norfolk House, Norfolk Street, Strand, W.C., and arrive there not later than Tuesday, June 14th, in time to appear in The Hospital which will be published on June 18th.

